# Birth outcomes in Colorado's undocumented immigrant population

**DOI:** 10.1186/1471-2458-5-100

**Published:** 2005-10-04

**Authors:** Mary M Reed, John M Westfall, Caroline Bublitz, Catherine Battaglia, Alexandra Fickenscher

**Affiliations:** 1 Department of Neurology, University of Colorado at Denver and Health Sciences Center, Denver, CO, USA; 2 Department of Family Medicine, University of Colorado at Denver and Health Sciences Center, Denver, CO, USA; 3 The Joffit Group, Inc., Denver, CO, USA; 4 American Indian and Alaska Native Program, Department of Psychiatry, University of Colorado at Denver and Health Sciences Center, Denver, CO, USA

## Abstract

**Background:**

The birth outcomes of undocumented women have not been systematically studied on a large scale. The growing number of undocumented women giving birth in the United States has important implications for clinical care and public health policy. The objective of this study was to describe birth outcomes of undocumented immigrants in Colorado.

**Methods:**

Retrospective descriptive study of singleton births to 5961 undocumented women using birth certificate data for 1998–1999.

**Results:**

Undocumented mothers were younger, less educated, and more likely to be single. They had higher rates of anemia, were less likely to gain enough weight, and less likely to receive early prenatal care. They were much less likely to use alcohol or tobacco. Undocumented women had a lower rate of low birth weight (5.3% v 6.5%, P < .001) or preterm infants (12.9% v 14.5%; p = .001). Undocumented women experienced higher rates of labor complications including excessive bleeding (2.3% v 0.8%, p < .001) and fetal distress (8.7% v 3.6%, p < .001).

**Conclusion:**

Undocumented women have lower rates of preterm delivery and low birth weight infants, but higher rates of pregnancy related risk factors. Higher prevalence of some risk factors which are amenable to medical intervention reveals the need for improved prenatal care in this group.

## Background

Studies of health status and health care in the undocumented immigrant population in the United States have been limited in both number and scope due to the transient and clandestine nature of the population, as well as the lack and inaccessibility of their medical records [1]. There are an estimated 7 million undocumented immigrants in the United States, growing 5% each year [2]. Birth certificate data is recorded for undocumented women who give birth in the United States; however, information regarding the mother's immigration status is not collected. Therefore, it is not possible to describe this population using birth certificate data alone. Previous studies of pregnancy and birth in this population have been limited to chart review in a single hospital setting,[3,4] analysis of proxy populations, like migrant women enrolled in Federal nutritional programs,[5,6] or by identifying the mothers by their country of origin[7].

Although the Personal Responsibility and Work Opportunity Act of 1996 bans undocumented immigrants form receiving most public benefits, they remain eligible for emergency medical services in life-threatening situations. Under the provisions of Emergency Medicaid, undocumented women qualify for coverage of labor and delivery services, provided that they meet all other eligibility requirements (including income). However, payor information is also not available on the birth certificate. Other researchers have successfully used billing data, specifically Medicaid claims, to identify births to women whose delivery services were covered by public funds [8].

Studies of Latin women of Mexican decent have demonstrated that immigrant groups tend to have healthier birth outcomes than would be expected from their socioeconomic profile, and that they are less likely to experience an adverse outcome with their first pregnancy than they are with subsequent pregnancies [9-11]. Studies of maternal characteristics and birth outcomes in migrant workers have shown that these women delay seeking prenatal care and gain significantly less weight during pregnancy than their non-migrant counterparts [6]. Recent studies have demonstrated an increase in incidences of neural tube defects in infants born to certain populations of Hispanic women who live along the Texas-Mexico border [12,13]. There has been no large systematic analysis of birth outcomes for undocumented immigrants.

The purpose of this study was to identify the birth outcomes for all undocumented women in Colorado for a 2-year period. Using Medicaid claims data linked to birth certificate data allowed anonymous identification of the infant birth record and description of maternal risks and birth outcomes in this population.

## Methods

This study linked Medicaid data to birth certificate data to isolate the cohort of undocumented women who gave birth in a Colorado hospital in 1998 and 1999. Emergency Medicaid is provided to undocumented non-citizens who are financially eligible for Medicaid. Emergency Medicaid only provides medical coverage for medical emergencies, which includes labor and delivery [14,15]. Emergency Medicaid does not provide coverage for routine prenatal care or out-patient post partum care. Colorado Medicaid adds the letter "J" to the identification number of all enrollees with Emergency Medicaid (EMJ). We obtained Medicaid records for all EMJ labor and delivery claims. Using a combination of non-unique identifiers, we merged the EMJ database with the Colorado Birth Record for the two-year period, 1998 to 1999. We used a previously described match-merge method[16] within SAS statistical software to merge the data sets on the basis of non-unique identifiers available in both datasets (infant's date of birth, date of hospitalization, maternal county of residence, maternal month and year of birth, and maternal country of birth). This method provides a robust dataset without identifying information on any individual patient. This study was reviewed and granted exempt status by the Colorado Multiple Institutional Review Board (Protocol 02-1048).

### Sample

All EMJ claims were abstracted from the total Medicaid claims database. All EMJ claims not related to delivery were eliminated (such as claims for preterm labor). The EMJ records were merged with the Colorado Department of Public Health and Environment's Birth Records birth certificate records for 1998–1999 using SAS statistical software's SQL procedure. The birth certificate record includes data on singleton and twin births. All twin and multiple birth certificate records were eliminated from analysis to avoid bias in low birth weight findings. Eighty-six percent of the EMJ delivery claims successfully matched to a single birth certificate. Approximately 1% of EMJ records matched multiple birth certificate records and 13% of EMJ records matched no birth certificate record. Of the 118,904 live singleton birth records, 5961 (5.01%) were matched to EMJ claims. Mexico was given as the mother's country of origin on ninety-three percent of the matched birth certificates.

### Measure and outcomes

Information obtained from the Colorado Vital Statistics Birth Record for this cohort was used to characterize known maternal risk factors before and during pregnancy, and birth outcome measures associated with higher risks of infant mortality or morbidity. Maternal risk factors predating pregnancy include maternal age, education level, marital status, and parity. Measures of maternal risks during pregnancy include adequate weight gain, number of prenatal visits, month of pregnancy when prenatal care began, and smoking or alcohol use.

Primary outcome measures included low birth weight, and gestational age. Secondary outcome measures included complications of labor and delivery, method of delivery, maternal medical risks, abnormal conditions of the newborn, and congenital anomalies of the child.

### Analysis

Chi-square tests were used for analysis of dichotomous maternal characteristics and pregnancy outcomes between undocumented women and all other women. The Student t-test statistic was used to analyze continuous variables of mother's age, infant's birth weight, and infant's gestational age. The Mantel-Haenzel test of association was used to determine if there was an association between low birth weight or preterm birth and undocumented status, while individually controlling for smoking status, weight gain during pregnancy, and age of the mother, the available variables known to have a significant impact on birth weight.

## Results

Maternal characteristics of the undocumented women were significantly different from the population as a whole (Table 1). Undocumented women were more likely to be between 17 and 35 years of age (93% v 87%, p < .001), less likely to have finished high school, and more likely to be unmarried than their counterparts in the general population.

**Table 1:** Maternal and pregnancy characteristics, Colorado singleton births, 1998–1999

**Maternal characteristics**	**Undocumented immigrants N = 5961 **(%)	**All other women N = 112,943 **(%)	**p-value**
**Demographics**			
Age			<.001
<17	3.1	2.2	
17–35	93.4	87.0	
>35	3.5	10.8	
Education: high school graduate	29.5	82.7	<.001
Not married	34.6	25.3	<.001
Parity			0.02
0	42.7	43.4	
1–2	47.5	47.9	
>2	9.8	8.7	
**Behavioral characteristics**			
Smoking	1.9	11.1	<.001
Alcohol Use	0.3	1.3	<.001
Weight Gain <20 lbs.	23.5	12.7	<.001
Number of Prenatal Visits			<.001
<9	47.3	20.1	
9–15	49.6	69.3	
>15	3.1	9.6	
Trimester Care Began			<.001
No prenatal care	1.7	1.0	
1	52.0	83.3	
2	34.3	12.9	
3	12.0	2.8	
**Medical risk factors**			
Anemia	7.7	2.2	<.001
Lung disease	0.8	0.5	.004
Gestational diabetes	2.7	1.7	<.001
Hypertension	2.9	3.2	0.23
Oligohydramnios	3.8	1.1	<.001
Previous >4000 gms birth	3.1	0.7	<.001
Previous preterm birth	2.1	1.3	<.001
No risk factors	51.5	76.0	<.001

Behavioral characteristics were also significantly different for the two groups. The undocumented women were far less likely to smoke (1.9 % v 11.1 %, p < .001). However, the undocumented women were less likely to have gained an adequate amount of weight during their pregnancy (23.5 % v 12.7 %, p < .001), and were less likely to receive an adequate number of prenatal care visits (47.3% v 20.1%, p < .001).

Undocumented women were less likely to have a primary (first time) C-section than other women in the population, and more likely to have a vaginal birth after C-section (Table 2). Undocumented women also experienced more complications of labor and delivery. They were significantly more likely to have meconium staining, excessive bleeding, precipitous labor, breech presentation, cord prolapse, and fetal distress (p < 0.01).

**Table 2:** Labor and delivery methods and complications; Colorado singleton births, 1998–1999

	**Undocumented immigrants N = 5961 **(%)	**All other women N = 112,943 **(%)	p-value
**Method of delivery**			
Vaginal delivery	80.1	81.7	0.17
Vaginal after C-Section	3.8	2.4	<.001
Primary C-Section	10.1	10.9	0.05
Repeat C-Section	5.1	4.9	0.56
Forceps/vacuum	9.7	8.6	<.01

**Complications of delivery**			
Meconium staining	11.2	4.3	<.001
Excessive bleeding	2.3	0.8	<.00
Premature rupture	1.9	2.3	0.06
Precipitous labor	2.4	1.8	<.01
Malpresentation	3.5	3.0	0.04
Cord prolapse	0.7	0.3	<.001
Fetal distress	8.7	3.6	<.001
No complications	60.1	73.5	<.001

The difference in mean birth weights was not clinically significant (Table 3). Undocumented women were significantly less likely to deliver a low birth weight infant (6.5% general population v 5.3% for undocumented women, p < 0. 001). The mean gestational age was slightly higher for the infants of undocumented women (39.1 weeks v. 38.9 weeks, p < .001). This difference is likely not clinically significant. However, the rate of preterm births was significantly lower among the undocumented group (12.9% v 14.5%, p < .001).

**Table 3:** Birth outcomes, Colorado singleton births, 1998–1999

**Newborn characteristics**	**Undocumented immigrants N = 5961**	**All other women N = 112,943**	
Mean birth weight	3268 gms	3250 gms	0.01
Very low birth weight (<1500 gms)	1.1%	1.0%	0.70
Low birth weight (<2500 gms)	5.3%	6.5%	<.001
Mean gestational age	39.1 weeks	38.9 weeks	<.001
Pre-term births (<37 weeks)	12.9%	14.5%	0.001
1-minute Apgar	7.68	7.73	<.01
5-minute Apgar	8.8	8.9	<.01
1-minute Apgar <5 (%)	7.8%	6.0%	<.001
5-minute Apgar <5 (%)	1.0%	0.7%	<.01
Abnormal conditions of the newborn	10.0%	7.8%	<.001
Congenital defects of the newborn	1.0%	1.2%	0.41

All abnormal conditions of the newborn (infant anemia, birth injury, fetal alcohol syndrome, hyaline membrane disease, seizures, and requirements for assisted ventilation) were collapsed into one category due to small numbers in individual cells. However, undocumented women showed significantly higher percentages than the general population in this combined category (10.0% v. 7.8%, p < .001). We did not find an increased rate of neural tube defects among infants of undocumented women in Colorado.

The Mantel-Haenzel test was used to assess the association of low birth weight and preterm birth with undocumented status, while individually controlling for the effects of smoking status, maternal age, or inadequate weight gain. The Breslow-Day test of the homogeneity of the odds ratio was not significant for any of the three analyses, and the common relative risk was estimated from the common odds ratio given by the Mantel-Haenzel test. Undocumented women were less likely to deliver a low birth weight infant even after controlling for smoking status (RR = 0.88, CI 0.79–0.99), weight gain (RR = 0.71, CI 0.63–0.80), and age (RR = 0.81, CI 0.72–0.90). Undocumented women were less likely to have preterm delivery even after controlling for smoking status (RR = 0.91, C.I. 0.85–0.98), weight gain (RR = 0.81, C.I. 0.75–0.87), and age (RR = 0.89, C.I. 0.84–0.96).

## Discussion

In this large statewide cohort of undocumented women we found lower rates of preterm delivery and low birth weight than the general population. Undocumented women had higher rates of maternal medical risks and less prenatal care. They had lower rates of tobacco and alcohol use. This is the first study to evaluate the birth outcomes of such a large, statewide cohort of undocumented women.

A 1992 study of maternal care coordination for migrant women in North Carolina cites several studies that document a high incidence of infant mortality and low-birth weight, as well as delay in seeking prenatal care [4]. The study was limited to one migrant health care center in the state, and the study population was comprised of only 599 female farm workers over a 5 year period. A Centers for Disease Control report on pregnancy related behaviors among migrant farm workers enrolled in a Special supplemental Nutrition Program for Women Infants and Children found that more migrants delayed seeking prenatal care, and gained less weight than non-migrant women [5]. However, prevalence was similar for low birth weight and preterm birth in the two populations. Although this was a four-state study, it was limited to women who were enrolled in the Nutrition program (N = 4840).

Studies of Latin women of Mexican decent have revealed at least two persistent differences in birth outcomes from the general population. In what has become known as the "Epidemiological Paradox", studies have repeatedly shown that some immigrant groups tend to have healthier birth outcomes than would be expected given their socioeconomic profiles.

However, children of immigrants do not seem to enjoy the same positive reproductive outcomes as their parents. This "acculturation effect" suggests that as immigrants become more Americanized, their risk of delivering a low-birth weight infant increases [9-11]. Populations of Mexican decent who reside along the Texas-Mexican border have been shown to have higher than average occurrences of neural tube defects. Furthermore, folic acid supplement in the Mexican-born Hispanic population along the Texas-Mexico border showed only a modest risk reduction in the incidence of neural tube defects [12,13].

Studies that have focused exclusively on the undocumented immigrant women have been limited to chart reviews at a single hospital, or survey data collected after using census data to identify areas likely to have a high immigrant population. One such study reported that fewer than 10% of illegal immigrants ever enroll in Medicaid, an indication of their reluctance to use government programs due to their fear of deportation [7].

Only about half of the undocumented women began prenatal care in their first trimester, as compared to almost 85 percent of the general population. The fact that they are residing in the United States illegally creates an obvious barrier to access to care. Because they often work at low paying jobs, they are frequently subject to substandard living conditions, and delay seeking professional medical advice regarding nutrition or prenatal care. Our study confirms these previous findings. The proportion of undocumented women who gained less than the Institute of Medicine's recommended weight was nearly twice that of the general population of pregnant women [17].

The effect of prenatal care has been measured largely in terms of its influence on low birth weight and preterm birth rates [18]. Our study showed significant differences in the proportions of infants born to undocumented women who experienced abnormal conditions at birth, as well as maternal complications during delivery and medical risk factors for the mothers. Better monitoring of both fetus and mother during pregnancy may reduce the risk of many adverse outcomes in addition to low birth weight and preterm birth. For example, medical assessment of the pregnant woman can detect risk factors such as gestational diabetes, anemia, and acute and chronic lung disease that may be amenable to intervention. Cohort studies of diabetic women have shown that control of the diabetes before conception can reduce the risk of congenital anomalies, which is a much as three times higher than for infants of non-diabetic mothers. The cause of anemia in pregnancy is almost always iron deficiency, and treatment with iron supplementation is generally effective.

One contributing factor to the finding that undocumented women were at reduced risk for delivering infants of low birth weight may be that they are much less likely to smoke or use alcohol. This advantage may be partially outweighed by the likelihood that they are less likely to gain the recommended amount of weight during their pregnancy, and that they postpone seeking prenatal care until later in their pregnancy. Our analysis shows that the undocumented women's reduced risk for delivering a low birth weight or preterm infant persisted after controlling either for maternal age or smoking history. Therefore, the reduced risk for low birth weight and preterm births for this group must be due to some factor (or factors) other than age, tobacco use, and inadequate weight gain.

The lack of prenatal care results in missed opportunities to monitor and prepare for labor and delivery, prepare for potential complications like malpresentation and placenta previa, or detect other pregnancy complication such as fetal anomalies and amniotic fluid abnormalities. Many complications of pregnancy and delivery may be avoided simply by assessing a woman's reproductive history [19]. While the outcome of low birth weight was lower among undocumented women, improved access to prenatal care could address the numerous other risk factors related to poor birth outcomes and might lead to even better outcomes in this population.

The major limitation to this study is the technique of merging distinctly different databases without unique identifiers. There were a significant number of EMJ files that did not match to a birth record and some that matched to more than one birth record. However, there is no clinical reason why matched records might systematically differ from unmatched records. The unmatched and multiple matched records were not included in the analysis. Birth certificate records are often incomplete, particularly in the areas of maternal complications and birth defects. However, birth certificate records are widely available, very accurate in terms of delivery method and birth weight and represent the standard measure for research on birth outcomes. The strength of this study was the creation and analysis of a database of a large cohort of women and infants that has previously gone unstudied. This method could be used in other states to evaluate the birth outcomes among undocumented women and to evaluate the impact of interventions to improve access and pregnancy management. The matching technique we used has been previously used and validated, and our matching rate was well within the range of previous studies [8,16].

## Conclusion

This study demonstrated that undocumented women are unique in terms of pregnancy risks among Colorado women, and their infants have characteristics that differ from the general population of newborns in the state. Undocumented women have more favorable birth outcomes despite receiving less prenatal care. Lower smoking rates may explain a large proportion of this difference. Focused care to improve access to early pregnancy care and diagnosis and treatment of common medical conditions such as anemia, gestational diabetes, and lung disease, may further help improve birth outcomes in this group.

## Competing interests

The author(s) declare there are no competing interests.

## Authors' contributions

John Westfall, MD, MPH, supervised all aspects of the study and assisted with the analysis, interpretation, and review of the manuscript. Catherine Battaglia RN, MS, PhD, conceived of the study and obtained the initial Medicaid billing dataset. Alexandra Fichenscher, MPH, assisted in the design and implementation of the study. Mary Reed, MPH, synthesized the analysis, assisted in the interpretation, and led the writing. Caroline Bublitz, MS performed all statistical analysis and assisted in the interpretation of the findings.

## Pre-publication history

The pre-publication history for this paper can be accessed here:


